# Cerebellar Transcranial Direct Current Stimulation (tDCS), Leaves Virtual Navigation Performance Unchanged

**DOI:** 10.3389/fnins.2019.00198

**Published:** 2019-03-12

**Authors:** Roberta Ferrucci, Silvia Serino, Fabiana Ruggiero, Claudia Repetto, Desirée Colombo, Elisa Pedroli, Sara Marceglia, Giuseppe Riva, Alberto Priori

**Affiliations:** ^1^Aldo Ravelli Center for Neurotechnology and Experimental Brain Therapeutics, Department of Health Sciences, International Medical School, University of Milan, Milan, Italy; ^2^Neurophysiology Unit, IRCCS Ca’ Granda Foundation Maggiore Policlinico Hospital, Milan, Italy; ^3^Neurologia I, ASST Santi Paolo e Carlo, Milan, Italy; ^4^Department of Psychology, Università Cattolica del Sacro Cuore, Milan, Italy; ^5^IRCCS, Istituto Auxologico Italiano, Applied Technology for Neuro-Psychology Lab, Milan, Italy; ^6^Department of Basic Psychology, Clinic and Psychobiology, Universitat Jaume I, Castellón de la Plana, Spain; ^7^Department of Engineering and Architecture, University of Trieste, Trieste, Italy

**Keywords:** cerebellum, cerebellar tDCS, spatial navigation, allocentric, egocentric

## Abstract

Spatial cognition is an umbrella term used to refer to the complex set of abilities necessary to encode, categorize, and use spatial information from the surrounding environment to move effectively and orient within it. Experimental studies indicate that the cerebellum belongs to the neural network involved in spatial cognition, although its exact role in this function remains unclear. Our aim was to investigate in a pilot study using a virtual reality navigation task in healthy subjects whether cerebellar transcranial direct current stimulation (tDCS), a non-invasive technique, influences spatial navigation. Forty healthy volunteers (24 women; age range = 20–42 years; years of education range 13–18) were recruited. The virtual reality spatial navigation task comprised two phases: encoding, in which participants actively navigated the environment and learned the spatial locations for one object, and retrieval, in which they retrieved the position of the object they had discovered and memorized in the previous encoding phase, starting from another starting point. Participants received tDCS stimulation (anodal or sham according to the experimental condition they were assigned to) for 20 min before beginning the retrieval phase. Our results showed that cerebellar tDCS left the accuracy of the three indexes used to measure effective navigational abilities unchanged. Hence, cerebellar tDCS had no influence on the retrieval phase for the spatial maps stored. Further studies, enrolling a larger sample and testing a different stimulation protocol, may give a greater insight into the role of the cerebellum in spatial navigation.

## Introduction

Over the past years, the concept of non-motor cerebellar functions has gathered growing attention becoming a reliable focal point for neuroscience investigators.

The cerebellum receives and sends information from various brain regions, cerebellar function is strongly associated with the hippocampus, and the cerebellum influences hippocampal activity, including spatial navigation (Rochefort et al., 2011; Rondi-Reig et al., 2014; Lefort et al., 2015). Information about the influence of the cerebellum on the navigation system comes from studies using electrophysiological, anatomical, and behavioral analyses in both human and animal models (Rochefort et al., 2013).

Ample information on the cerebellum’s spatial function comes from experiments conducted on rats, mice and goldfish (Petrosini et al., 1996; Durán et al., 2014). Studies involving different cerebellar mutant mice strains or using hemi-cerebellectomy combined with widely ranging protocols have reported selective deficits in these animals’ spatial functions. Some studies have proposed that this impairment is related more to the procedural navigational component (inability to organize and execute complex and effective exploration behaviors) than to the declarative component (an inability to develop an internal environment map).

Earlier findings suggesting that the cerebellum influences path integration, a function that incorporates proprioceptive and vestibular information as a subject moves through the environment (McNaughton et al., 2006). Rather than encoding a spatial map of the environment the cerebellum computes self-motion information from the various sources required to build the body’s representation in space (Rochefort et al., 2013). The cerebellum contributes to spatial navigation at two levels, first in processing the self-motion information required to build spatial representation, and second in using this spatial representation to perform an optimal trajectory toward a goal. The cerebellar network participates in map formation in the forebrain navigation areas by specifically encoding and computing self-motion information(Rochefort et al., 2013).

Over recent years, evidence involving the cerebellar region in non-motor functions has made it of interest as a target for electrical neuromodulation, intended to alter the acquisition of spatial knowledge and skills (Brunyé et al., 2014; Oldrati et al., 2018).

One of the most often used virtual reality tasks for investigating spatial navigation tasks comprises two phases: encoding, in which participants actively navigate the environment and learn the spatial locations for one object, and retrieval, in which they retrieve the position of the object they have discovered and memorized in the previous encoding phase, starting from another starting point. Specifically, this “re-orientation task” forces participants to refer to the long-term stored allocentric environmental representation and synchronize it with new upcoming egocentric inputs to solve the task, thus making it an effective procedure for assessing egocentric-to-allocentric translation ability (Bosco et al., 2008; Serino and Riva, 2015).

To our knowledge, few researches have addressed transcranial direct current stimulation (tDCS) and spatial navigation in a virtual environment (Brunyé, 2018; Brunyé et al., 2018) and no published studies have studied the effect of cerebellar tDCS. Having his information would help us to understand more about the possible role of the cerebellum in spatial navigation thereby advancing research to develop better cognitive treatment outcome measures (Coughlan et al., 2018) also for use in patients with neurological syndromes who have deficits in spatial navigation and orientation.

In this pilot study, we used a virtual navigation task in healthy subjects to investigate whether cerebellar tDCS influences spatial navigation.

## Materials and Methods

### Participants

Complying with the generally recommended sample size of 10 to 40 participants for a pilot study (Whitehead et al., 2016), we recruited 40 volunteers (24 women; mean age = 26.65, mean years of education = 17.05). Participants had normal or corrected-to-normal vision and no history of psychiatric or neurological illnesses, nor they were taking medication affecting the central nervous system. All the volunteers showed normal visuospatial learning abilities compared with the reference population, as assessed by the Corsi Supra-span test (Spinnler, 1987). All the participants signed written informed consent. The study was approved by the institutional review board and conducted in accordance with the Declaration of Helsinki.

### Procedure

Participants were randomly assigned to one of two (between subjects) conditions: real stimulation or sham. As a first step, participants underwent a cognitive assessment to evaluate attention (simple visual reaction times, RTs) task (Barbarotto et al., 1998) and visuospatial learning (Corsi Supra-span learning). Afterward, they did the virtual task. Participants stood in front of the computer, wore a head mounted display (HMD) and held the joypad with both hands. When they confirmed feeling comfortable with the technology set up, they did a simple navigation task (training phase). Once they could manage the task, the encoding phase started. It usually took only a few minutes (up to 3). After that, participants sat down comfortably on a chair and received tDCS stimulation (real or sham according to the experimental condition they were assigned to) for 20 min. The retrieval phase in the virtual task began 30 min after stimulation ended. We used this time delay for studying the effect of cerebellar tDCS in spatial navigation because our previous study (Ferrucci et al., 2008) showed that the major effect became evident 35 min after cerebellar tDCS ended.

**FIGURE 1:**
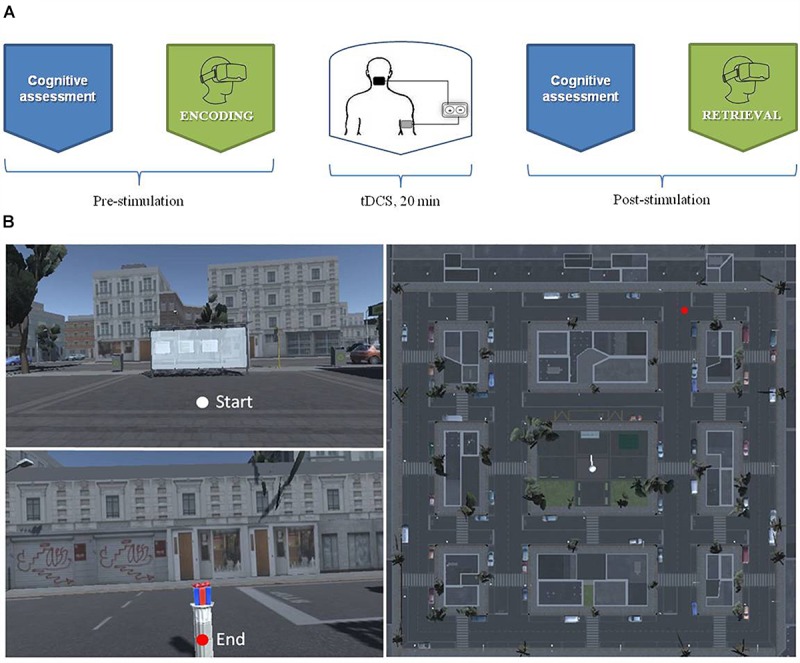
**(A)** The experiment procedure over the timeline **(B)**: The aerial view of the virtual city.

During the pause, participants repeated the cognitive assessment. Each experiment lasted about 1 h and 30 min (Figure 1A).

### The Virtual Task

Using the software Unity3D^1^ we developed a virtual city corresponding to a small district measuring 150 by 150 m in size, built in a regular grid pattern around a central square (Figure 1B). The central square comprised a bus stop, a newsstand, several flower beds and benches. Throughout the city, buildings, shops and cars were parked alongside the streets. No human beings were present in the environment. The virtual task was delivered using a portable computer, connected to a joy pad that allowed participants to explore the virtual city. Participants visualized the environment with the HMD (Oculus Rift DK2) which made the experience fully immersive. Participants could rotate the head to explore the surrounding environment. They were also required to rotate the whole body to change the navigation direction (as in the real world).

During the “training phase,” participants familiarized themselves with the virtual reality technology performing a simple navigation task. In the “encoding phase,” starting from the bus stop positioned in the central square, participants were explicitly required to find and then to memorize the position of a hidden object (i.e., gift box). They were therefore free to explore the virtual city with no time limits but were told that the time would be recorded to avoid excessive hesitation. They were also told that their task was to find the gift box placed somewhere within the city, as fast as possible.

In the “retrieval phase,” participants began the navigation from another starting point, diametrically opposite to that in the encoding phase. This new starting position was located at the same distance away from the object as the previous one (during the encoding phase). Participants were invited to retrieve the position of the object (i.e., now absent) they had discovered and memorized in the previous phase. Also in this experiment, participants were given no time limit, but time was recorded, so they were asked to work as fast as possible.

### Cerebellar Transcranial Direct Current Stimulation

Cerebellar tDCS was delivered with an electrical constant direct current stimulator (HDCstim, Newronika, Italy) connected to a pair of a rectangular saline-soaked synthetic sponge electrodes (6 × 7 cm). To avoid confounding biases arising from 2 electrodes with opposite polarities over the scalp, we used a non-cephalic reference electrode (Ferrucci et al., 2008, 2013, 2015; Ferrucci and Priori, 2014). The active electrode was centered on the median line 2 cm below the inion with its lateral borders about 1 cm medially to the mastoid apophysis (over the cerebellum) and the reference electrode over the right deltoid muscle. The stimulus was either an anodal current at 2 mA intensity (current density: 0.06 mA/cm^2^) or a sham current (placebo) delivered for 20 min over the cerebellum (Ferrucci et al., 2015). After a short-lasting and mild itching sensation at both electrodes in the first 10/20 s, subjects perceived no other sensation during tDCS.

For sham tDCS the electrodes were placed as for real stimulation and the stimulator was turned on for 20 s, so that the subjects felt the initial itching sensation, as they did during anodal tDCS, but thereafter received no current.

### Data Analyses and Statistical Approaches

Three accuracy indexes were used to measure effective navigational abilities.

#### Offset

Calculated as the distance (in meters) between the gift box point as estimated during the retrieval phase, and the exact point where the gift box was placed in the encoding phase.

#### TimeRet

The duration (in seconds) of navigation during the retrieval phase. It measured the time participants took to identify the object’s location.

#### DistanceRet

The distance (in meters) traveled during retrieval; it measured how long the environment exploration lasted before participants thought they had identified the correct object position.

Data were analyzed with SPSS (v 24.0) and JASP (Version 0.8.5). Because they violated the normality assumption, all indexes were log-transformed before being entered into the database. First, each accuracy index was analyzed with separated independent sample *t*-tests. After that, to understand whether possible confounding factors influenced the tDCS-induced effects, a series of univariate analyses of covariance (ANCOVAs) were run, with each of the accuracy indexes as dependent variables, stimulation (real vs. sham) as between subject’s variable and Corsi Supra-span score (at baseline assessment) as covariate, to check whether the individual visuospatial learning abilities influenced the navigation performance. To investigate whether cerebellar stimulation influenced attention abilities repeated measures ANOVAs with the RTs as dependent variables, the time as within-subject factor with two levels (Pre vs. Post-Stimulation), and Stimulation as between-subjects factor (Real vs. Sham).

In addition, we also used a different statistical approach based on Bayesian analysis, a procedure that is more suitable for assessing null experimental effects (Jarosz and Wiley, 2014). To compute a ratio between the likelihood of the data given the null-hypothesis and given an alternative hypothesis (Nuzzo, 2014; Rouder, 2014), we tested each of the accuracy indexes with the paired sample *t*-test Bayes factor (BF).

## Results

No differences were found between the two groups for age (*t*_38_ = 1.31; *p* = 0.2), education (*t*_38_ = -0.41; *p* = 0.2), and gender (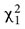 = 0.52; *p* = 0.75) (Table 1). Independent sample *t*-tests underlined that accuracy indexes remained unchanged regardless of the type of stimulation (Offset: *t*_38_ = -1.32; *p* = 0.2; *d* = 0.366; DistanceRet: *t*_38_ = 1.22; *p* = 0.23; *d* = 0.126; TimeRet: *t*_38_ = 1.2; *p* = 0.24; *d* = 0.118) effect sizes of Cohen’s d equal to 0.2, are considered small, 0.5 considered medium, and 0.8 considered large) (Cohen, 1988). The univariate ANCOVAs showed that the individual visuospatial learning abilities had no effect on the navigation performances (Offset: *F*(1.37) = 0.05; *p* = 0.83; η^2^ = 0.001; DistanceRet: *F*(1.37) = 0.04; *p* = 0.85; η^2^ = 0.001; TimeRet: *F*(1.37) = 0.36; *p* = 0.55; η^2^ = 0.01). Similarly, stimulation induced no changes in attention abilities (RTs: Time X Stimulation *F*(1.38) = 0.06; *p* = 0.81; η^2^ = 0.002).

**Table 1:** Data for reaction times (RT) and accuracy indexes.

		Mean	Median	St. Dev.	Min	Max
**Descriptive statistics**		
RT_pre	*Sham*	309.88	301.94	35.75	263.97	381.11
	*Real Stimulation*	311.88	310.07	20.12	274.51	357.51
RT_post	*Sham*	316.74	312.58	27.45	283.28	376.40
	*Real Stimulation*	320.95	314.44	30.04	275.40	398.40
DistanceRet	*Sham*	242.42	194.21	169.46	82.49	692.51
	*Real Stimulation*	218.83	153.47	203.56	13.21	804.65
Offset	*Sham*	34.88	7.20	40.21	1.10	105.67
	*Real Stimulation*	50.41	41.67	44.58	1.94	117.04
TimeRet	*Sham*	142.30	108.00	84.24	55.00	350.00
	*Real Stimulation*	130.80	97.50	108.37	7.00	431.00

		**BF01**			**error%**	

**Bayesian independent sample *T*-test factors for all the accuracy indexes**						
DistanceRet		1.802			0.008	
Offset		1.619			0.007	
TimeRet		1.841			0.008	


The second statistical approach based on Bayesian analyses confirmed that cerebellar tDCS left the accuracy indexes unchanged. The estimated Bayes factors (null/alternative) indicated that the data would fit better under the null hypothesis than under the alternative one, 1.8 times for the DistanceRet, 1.62 times for Offset and 1.84 times for TimeRet (Table 1). In accordance with recommendations by Wetzels et al. (2011), the obtained Bayes Factor indicated anecdotal evidence (Table 1).

## Discussion

Our findings in this pilot study show that in healthy subjects, cerebellar tDCS leaves the retrieval phase in the virtual navigation task – a measure that reflects the spatial navigation system – unchanged.

The first researchers to identify the crucial role of the cerebellum in the motor and cognitive aspects of navigation were Igloi et al. (2015) using a virtual reality task, highlighting a functional interaction between the cerebellum and the hippocampus to support spatial encoding. As recently reviewed by Rochefort et al. (2013), the cerebellum may specifically contribute to two major brain circuits supporting the representation of space in the hippocampal system: one involving the retrosplenial cortex, responsible for the egocentric-allocentric transformation (Vann et al., 2009); and the other involving the posterior parietal cortex, responsible for egocentric representations (Taube, 2007).

Of importance when interpreting our results, we emphasize that our individual participants’ navigation experiences differed markedly in quantity and quality. Although during navigation tasks, subjects probably use two different strategies, one egocentric and the other allocentric (Serino and Riva, 2015), we did not explore the relationships between these abilities with quantitative measures. We conjecture that in our study, during the retrieval phase, subjects used allocentric strategies more extensively than egocentric strategies whereas in the “encoding” phase during the virtual navigation task, they used an egocentric learning spatial strategy based on praxis strategies, related to procedural learning (Schenk and Morris, 1985).

Because the cerebellum is primarily involved in acquiring procedural aspects of the spatial tasks whereas the hippocampus is more involved in recollecting previous episodic spatial experiences (Petrosini et al., 1996; Burgess et al., 2002; Valenzuela et al., 2003), whether stimulation delivered before the encoding phase might be more effective than stimulation delivered before the retrieval phase remains to be tested in a specifically designed experiment.

Why cerebellar tDCS failed to influence navigation more effectively could also depend on several factors including the type of stimulation delivered (anodal-cathodal). Because cerebellar tDCS studies have assessed neurophysiological, cognitive, affective, as well as behavioral variables with heterogeneous methodologies, interpreting the effects induced by anodal or cathodal stimulation is a far more complex task for cerebellar tDCS than for cerebral tDCS. Equally important, the stimulation site (unilateral or bilateral cerebellum), reference electrode position and stimulation variables (intensity, duration, electrode size) differed across the various studies (Ferrucci and Priori, 2014; Ferrucci et al., 2015; van Dun et al., 2017; Oldrati and Schutter, 2018). Several methodological variables for cerebellar tDCS therefore need to be systematically assessed. Cerebellar tDCS remains poorly understood and in general, no clear reference standards exist for tDCS dose and montage.

This study has some limitations. First is the small sample size, though large enough for a pilot study, and the inter-individual variability. Another limitation is that we delivered cerebellar tDCS only before the retrieval phase.

Further studies enrolling a larger sample and testing a different stimulation protocol in another virtual reality task phase may be useful in understanding better the cerebellum’s specific role in spatial navigation.

## Author Contributions

RF conceived and performed the experiments, analyzed the data, and wrote the paper. SS, FR, and CR conducted the experiments, analyzed the data, and wrote the paper. DC and EP conducted the experiments. SM analyzed the data and wrote the paper. GR and AP conceived the experiments, coordinated the research, and reviewed the paper. All authors critically revised and approved the final manuscript.

## Conflict of Interest Statement

RF, SM, and AP are founders and stakeholders of Newronika srl, which is a spin-off company of the University of Milan and of the Fondazione IRCCS Ca’ Granda Ospedale Maggiore Policlinico. AP is on the scientific advisory board of the same Newronika srl spin-off company. The remaining authors declare that the research was conducted in the absence of any commercial or financial relationships that could be construed as a potential conflict of interest.

## Footnotes

1 www.unity3d.com
